# Supplementary material for: Learning-Induced Shifts in Mice Navigational Strategies Are Unveiled by a Minimal Behavioral Model of Spatial Exploration
Source: eNeuro. 2021 Sep 29;8(5):ENEURO.0553-20.2021. doi: 10.1523/ENEURO.0553-20.2021 (PMC8489025; doi:10.1523/ENEURO.0553-20.2021)
Supplement: Extended Data 1 — HexMaze behavior analysis. Download Extended Data 1, ZIP file. [file enu-eN-NWR-0553-20-s03.zip › README.txt]

EXAMPLE CODE FOR ANALYSES PERFORMED IN THE PAPER: "Learning-induced shifts in mice navigational strategies are unveiled by a minimal behavioral model of spatial exploration"MATLAB code reproducing the paper analysis : - HexMaze_Code_Beh : Computes Relative Trial Length and Distance from Optimal Path as described in the paper.- HexMaze_Code_Sim : Compares statistical distribution of Relative Trial Length between experimental data and data obtained from modelling a virtual agent following a parametrised behaviour. The MATLAB .mat file ‘’HexMaze_ExData’ contains a sample of the experimental data (Build-Up, 1st Session) and can be used to run the code. It can also be used to get the input data format for the available code. 
